# Mathematical modelling of golden apple drying and performance evaluation of solar drying systems using energy and exergy approach

**DOI:** 10.1038/s41598-025-92133-2

**Published:** 2025-03-06

**Authors:** Halefom Kidane, Istvan Farkas, Janos Buzás

**Affiliations:** 1 Doctoral School of Mechanical Engineering, Hungarian University of Agriculture and Life Sciences, Pater K. u. 1, Gödöllő, H-2100 Hungary; 2 Institute of Technology, Hungarian University of Agriculture and Life Sciences, Páter K. u. 1, Gödöllő, H-2100 Hungary

**Keywords:** Drying, Energy, Exergy, Falling period, Thin layer, Solar energy, Engineering, Mathematics and computing

## Abstract

The study presents the energy and exergy analysis of two differently shaped dryers connected to the two same type of drying system and drying kinetics of the apple slice dried in the stated drying chambers. The exergy inflow, outflow and efficiency of the solar air hears and drying chamber were evaluated. The result revealed that significant variations in energy and exergy efficiencies were observed within the dryers and solar air heaters (SAHs). The average efficiency of SAH 1 and SAH 2 on day 1 were 53.85% and 49.79%, respectively, while the corresponding dryers’ average efficiencies were 37.94% and 36.15%. On day 2, the average efficiency of SAH 1 and SAH 2 were 47.18% and 44.19%, respectively, with the dryers’ average efficiencies of 35.9% and 34.4%. The average exergy efficiency followed the same pattern as energy efficiency, with SAH 1 and Dryer 1 being higher than SAH 2 and Dryer 2 on both days. The drying of apple slices took place entirely during the falling rate period. To predict drying behavior of the apple slices, ten thin-layer drying models were assessed. The Midilli et al. was found the best fit (R^2^ = 0.9969), followed by Logarithmic and Two-term models.

**Supplementary Information:**

The online version contains supplementary material available at 10.1038/s41598-025-92133-2.

## Introduction

Drying plays a crucial role in extending the shelf life of food and agricultural products by reducing their moisture content to a stable level, typically below 10% on a wet basis. However, the high energy demands of this process can make it expensive and economically difficult for end users, which limits the popularity of traditional or electricity-powered drying methods. Such challenges can be alleviated by adopting alternative energy sources, particularly solar energy^1^. As reported by Obaideen et al.^2^ for decades, renewable energy has been recognized as a key solution to the global energy crisis.

Solar energy is a plentiful, cost-free, and sustainable power source that has been utilized in various applications. As a clean and renewable alternative to conventional energy sources, it holds significant promise. For low-temperature applications, such as food drying, solar energy is considered the most effective option^3^. As noted by Barghi et al.^4^, the solar cabinet dryer is the most widely used type of solar drying system. In this drying method, the products are kept out of direct sunlight, and the thermal energy sources are separated from the items being dried. This design helps preserve the consistency and color of the products. Furthermore, drying multiple crops simultaneously can take longer with this system.

Evaluating the performance of solar drying systems and analyzing the drying behavior of products are essential for enhancing efficiency, maintaining product quality, optimizing energy consumption, and driving advancements in the design of solar drying technologies. Researchers have employed various approaches to analyze the physical performance, thermal characteristics, and drying behavior of products in indirect-type solar drying systems. Among these, energy and exergy analyses are the most commonly used methods to evaluate the efficiency of solar drying systems. Here are some recent studies that utilized energy and exergy analysis to evaluate their drying system and analysis the drying kinetics of agricultural products. For example, Barghi et al.^4^ investigated solar drying chamber with a porous plate and phase change material connected to unglazed transpired collector connected to a dryer used Teucrium podium as dried samples, Singh et al.^5^ analyzed indirect solar dryer used wheat seeds as the dried sample. Fudholi et al.^6^ studied an indirect solar drying system with a double-pass solar collector and finned absorber used red chili, Şevik et al.^7^ examined double-pass solar air dryer (DPSAD) and an infrared-assisted double-pass solar air dryer (DPSAIRD) dried apple slices and mint leaves, Rabha et al.^8^ analyzed ghost chilli pepper and ginger with an indirect-type forced convection solar tunnel dryer, Sethi et al.^9^ studied potato chips using an V-groove assist rotating tray type solar cabinet dryer, Mugi et al.^10^ carried out an experiment in an indirect solar dryer integrated with a trapezoidal duct used okra as dried sample, Tagnamas et al.^11^ analyzed an indirect convective solar dryer and tested carob pulp, Boulemtafes-Boukadoum and Benzaoui^12^ carried out an experiment in indirect solar drying chamber and they used mint as dried sample.

Bhardwaj et al.^13^ tested medicinal herbs in solar dryer integrated with sensible heat storage material and phase change material, Sharma et al.^14^ evaluated an indirect-type domestic hybrid solar dryer and characterize drying kinetics of tomatoes, Rao and Sivalingam^15^ studied Krishna Tulsi using an evacuated tube solar dryer, Dash et al.^16^ investigated an indirect-type flat plate collector solar dryer to dry black cardamom, Idlimam et al.^17^ examined forced convection solar dryer to dry dandelion root and Subramaniyan et al.^18^ assessed groundnut using an indirect solar dryer with forced convection solar dryer.

The study deals with analysis of energy and exergy was conducted for two differently shaped drying chambers which used apple as dried sample, each connected to similarly shaped solar air heaters. The novelty of this article lies in the fact that no prior research has conducted energy and exergy analysis, along with mathematical modeling, of apple slices using two differently shaped drying chambers connected to similarly designed solar air heaters. This study presents a comprehensive exergy analysis that highlights significant variations in energy inflows and outflows between two drying systems. Additionally, the study explores the mathematical modeling of golden apple drying using two different solar drying chambers, enhancing the accuracy of drying kinetics for the product under consideration. The findings emphasize the critical role of mathematical modeling, along with energy and exergy analysis, in improving the efficiency and sustainability of solar drying systems.

## Materials and methods

### Study area

The study was carried out in the forecourt of the Mechanical Engineering Laboratory at the Hungarian University of Agriculture and Life Sciences (MATE) in Gödöllő, Hungary, during select sunny days in August and September 2024. The geographical coordinates of the location are 47° 35’ 39” N and 19° 21’ 59” E.

### Description experimental setups

The experimental setup consisted of several essential components designed to optimize the solar drying process. These included a solar air heater, drying chambers, fans, support structures, and a base. A custom-designed solar air heater with a diffuser-shaped inlet (1.25 m × 0.5 m) was fabricated in our laboratory. The solar collector was covered with a 4-mm-thick plexiglass sheet, which allowed sunlight to pass through while minimizing heat loss. Beneath the plexiglass, a 1.2-mm-thick copper absorber plate efficiently captured solar radiation, raising the temperature within the collector.

Two drying chambers, each equipped with four trays, were also fabricated. Each chamber measured 1 m in height, 0.5 m in width, and 0.5 m in length. To enhance thermal efficiency, the chambers were insulated with 5 cm thick extruded polystyrene (XPS) boards. This insulation material, with a density of 35 kg/m³, a specific heat capacity of 1450 J/(kg K), and a thermal conductivity of 0.033 W/(m K), significantly reduced heat loss during operation. The trays, made of fiberglass mesh, provided excellent airflow and ensured secure placement of the drying materials.

### Data collection procedure

The main materials and instruments utilized in the experiment are detailed in Table 1.

**Table 1:** List of sensors and instruments used during the experiment.

Instrument/Sensor	Model	Accuracy	Operational range	Quantity
Humidity sensor	HIH-40000-004	± 3.5%	0 -100% RH	6
Pressure sensor	SDP816-500 Pa	± 5 millibar	–50–500 Pa	2
RTD	PT-100	± 0.1 °C at 0 °C	-75ºC- 250ºC	8
Temperature recorder	BTM-4208SD	± 0.5 °C	**-**200 °C -+400 °C	1 with 12 channels
Digital anemometer	MS6252A	± 0.01 m/s	0.40–30.00 m/s	1
Vernier Caliper		-	-	1
Digital weighing machine	Lutron GM-500	0.1% + 2 digit	0–500 gram	1
Pyranometer	Kipp and Zonen MM11	± 0.1 W/m	1–4000 W/m²	1

**Fig. 1:**
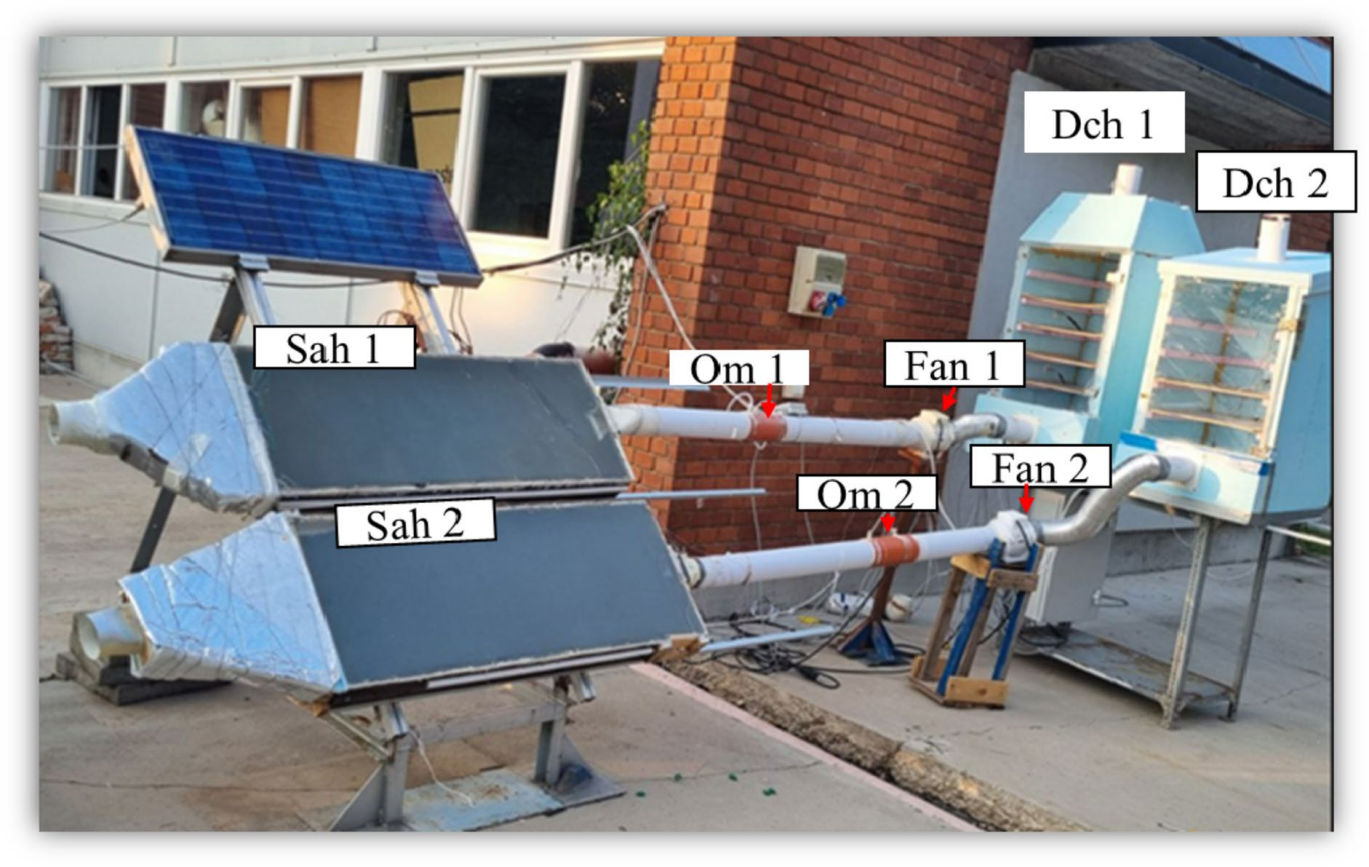
Solar drying chambers integrated with solar air heaters.

TT-100 types of fans with a minimum power consumption of 21 W or an airflow of 145 m³/h, and a maximum of 33–187 m³/h, were utilized to facilitate the forced intake of heated air was used. The complete assembly of the solar air collector, including the drying chambers, fans and connected tubes is depicted in Fig. 1.

**Fig. 2:**
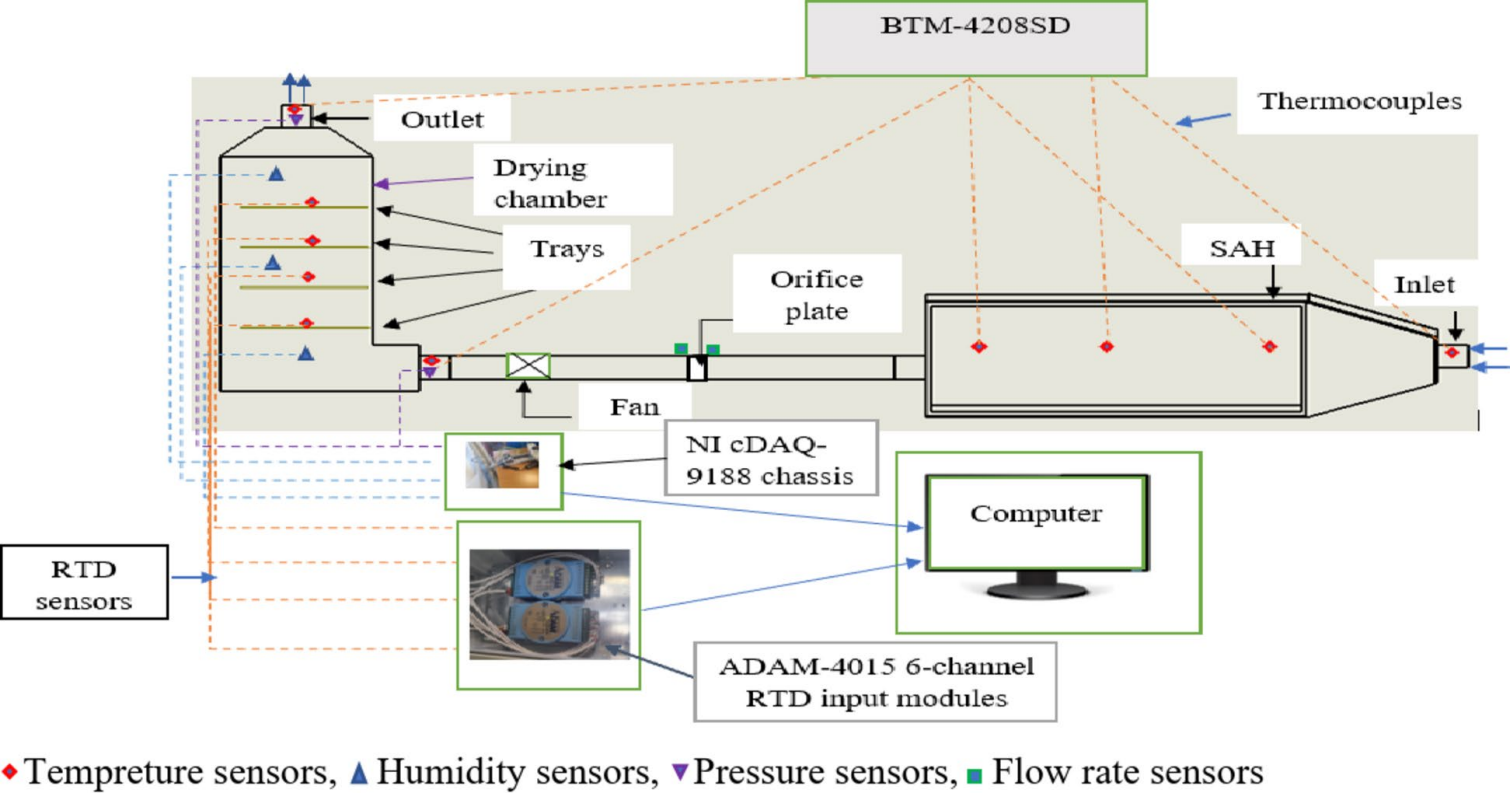
Arrangement of the sensors (side view).

The arrangement of the instruments and sensors is illustrated in Fig. 2.

The pressure drops (D_p_) of the SDP806/SDP816-500 Pa pressure sensor with square root configuration based on the company’s manual instruction can be calculated by following equation:

1 $$\text{D}_\text{p} \:\pm\:\left(\frac{{A}_{OUT}}{{V}_{DD}}-0.5\right){\left(\frac{{A}_{OUT}}{{V}_{DD}\cdot\:0.4}-1.25\right)}^{2}525$$

where $\:\pm\:$ is flow direction.

The mass flow rate was determined by the following expression^19^:

2 $$\:\dot{m}\:=\:\text{C}\text{d}\times\:\text{v}\times\:\text{A}\sqrt{2{{\times\:g}_{c}\times\:\rho\:}_{a}{\times\:D}_{p}}$$

For the humidity sensors, the following formulas were employed based on the manufacturer’s guidelines:

3 $${\rm{ Voltage\,output}} = \rm{V_{supply}}\left( {0.0062} \right){\rm{ }}\left( {sensor{\rm{ }}RH} \right){\rm{ }} + 0.16$$

4 $${\rm{Sensor\, RH}}\:\:\frac{{V}_{OUT}-0.16}{0.006\cdot\:{V}_{supply}}$$

5 $${\rm{True\, RH}}\:\:\:\:=\frac{Sensor\:RH}{1.0546-0.00216T}$$

### Sample preparation

**Fig. 3:**
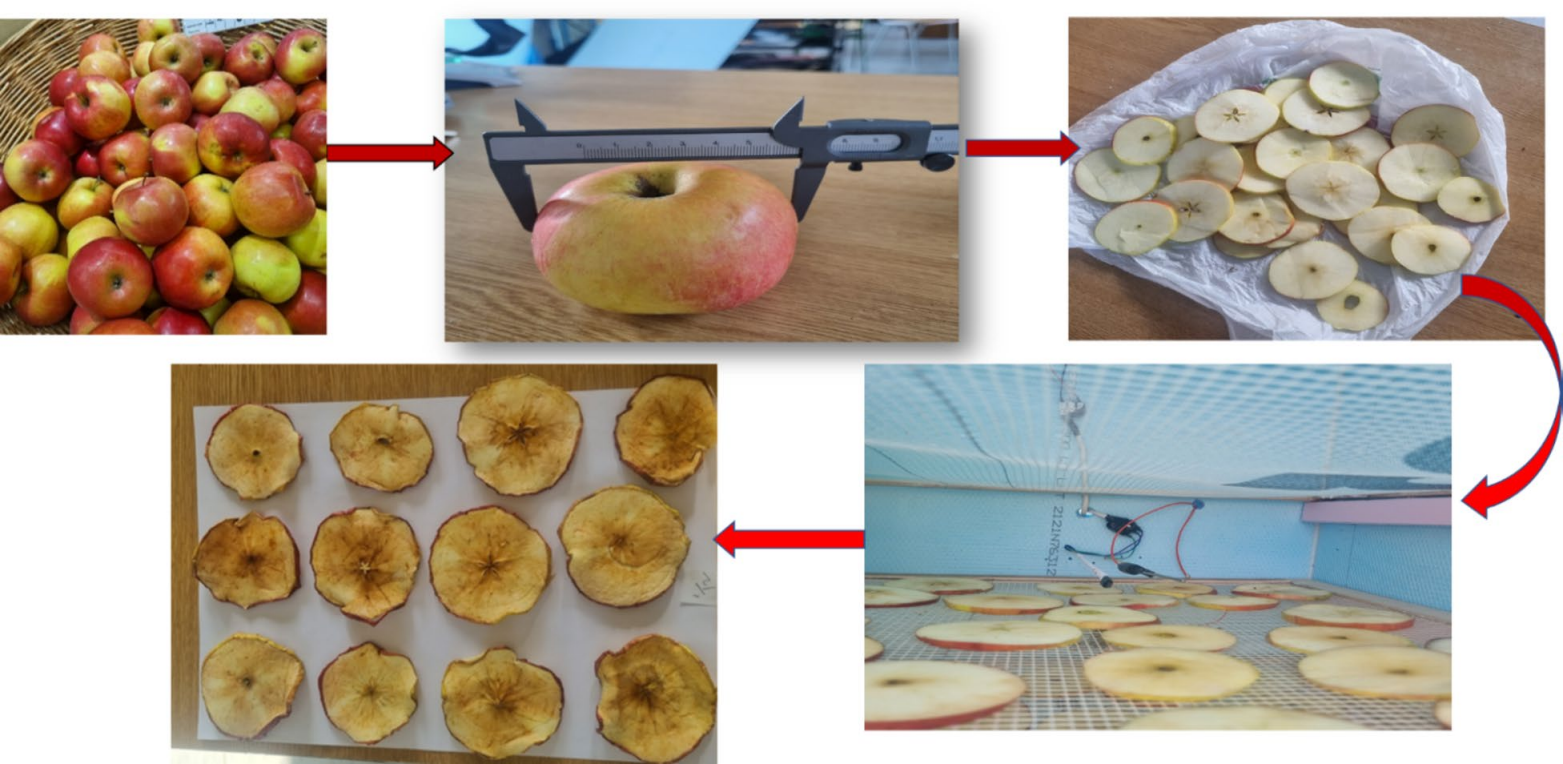
Sample preparation.

Three kilograms of golden apples were purchased from Coop, a local grocery store in Gödöllő city, Hungary. A random batch was selected, and two kilograms were carefully chosen based on size and external quality. The apples had diameters ranging from 0.75 m to 0.83 m. They were sliced horizontally into cylindrical pieces with thicknesses between 0.004 m and 0.006 m. Each slice was weighed to determine the necessary drying time. The slices were then placed on a tray in the drying chamber to begin the drying process. The basic sample preparation of the drying process used is shown in Fig. 3.

### Uncertainty of the experiment

Several factors lead to uncertainty in the experimental readings, including improper instrument selection, environmental influences, inaccurate measurements, and human error^20^.

Uncertainty analysis for the dependent parameters can be assessed as follows^21^:

6 $$\rm{U(y)}=\:\sqrt{{\left(\frac{\partial\:y}{{\partial\:x}_{1}}\right)}^{2}{u}^{2})\left({x}_{1}\right)+{\left(\frac{\partial\:y}{{\partial\:x}_{2}}\right)}^{2}{u}^{2})\left({x}_{2}\right)+\dots\:{\left(\frac{\partial\:y}{{\partial\:x}_{n}}\right)}^{2}{u}^{2}\left)\right({x}_{n})}$$

where y *i*s the dependent parameters and *x*_1_, *x*_2_, x_n_ measured value.

In our case the combined total uncertainty of all instruments used was approximately 3.59%, which is acceptable to conduct our experiment.

### Principle of energy and exergy analysis

Energy analysis in thermodynamic systems is crucial for understanding how to effectively utilize energy. In contrast, exergy analysis provides insights into the amount of energy available for performing useful work, while also highlighting losses that occur due to irreversibility in processes. By conducting thorough energy and exergy analyses, one can develop more efficient systems^8^. Energy and exergy analyses are crucial for understanding and improving the efficiency of drying processes in thermal systems. Exergy analysis, in particular, plays a key role in identifying energy losses and optimizing system design, making it a powerful tool for enhancing overall performance and achieving optimal drying conditions^5^.

### Energy analysis

The evaluation of a solar dryer’s energy performance is essential for enhancing its efficiency and thermal effectiveness. This analysis aids in determining optimal operating parameters and identifying necessary design enhancements, ensuring both environmental sustainability and economic feasibility^22^. Energy analysis in solar drying system involves applying the principles of mass and energy conservation, assuming a steady-state flow. The input energy to the ISD is derived from solar radiation, while the output energies include losses through gaps in the SAC and drying cabinet, as well as the energy of the air exiting the chimney^23^. The general equations representing the principles of mass conservation and energy conservation are outlined in the following equations.

General equation of mass conservation of drying air:

7 $$\:\sum\:\left({\dot{\text{m}}}_{\text{h}\text{u}\text{i}}+\dot{{\text{m}}_{\text{p}\text{i}}}\right)=\:\sum\:\left({\dot{\text{m}}}_{\text{h}\text{u}\text{o}}\right)$$

General equation of energy conservation:

8 $$\:\dot{\text{Q}}-{\dot{\text{W}}=\sum\:{\dot{\text{m}}}_{0}\left(\dot{\text{h}}_{\text{o}}+\frac{{\text{v}}_{\text{o}}^{2}}{2}+{\text{Z}}_{\text{o}}\text{g}\right)}-\sum\:{\dot{\text{m}}}_{\text{i}}\left({\text{h}}_{\text{i}}+\frac{{\text{v}}_{\text{i}}^{2}}{2}+{\text{Z}}_{\text{i}}\text{g}\right)$$

where, $\:\dot{Q}$ net heat transfer into the system (W) and $\:\dot{W}$ is the net work done by the system(W) h is enthalpy (J), v is velocity(m/s), and Z is height relative to a reference point (m)

This study examines various energy evaluation systems within the drying process.

### Energy analysis of solar air heater

There are various types of energy associated with solar air systems, but only the fundamental ones are discussed here as follows.

The heat obtained (Qu) from the solar air heater which is employed to elevate the temperature of the air inside the drying chamber^24^,is expressed as follow:

9 $${\rm{Qu}} =\:\:{\dot{\text{m}}}_{\text{a}}{\text{C}}_{\text{p}\text{a}}\left({\text{T}}_{{\text{S}\text{A}\text{H}}_{\text{o}}}-{\text{T}}_{\text{a}\text{m}\text{b}}\right)$$

The efficiency of the solar air heater (η_SAH_) is computed by employing the following Eq. ^23^:

10 $$\:{{\upeta\:}}_{\text{S}\text{A}\text{H}}=\frac{{\dot{\text{m}}}_{\text{a}}{\text{C}}_{\text{p}\text{a}}\left({\text{T}}_{\text{S}\text{A}\text{H}\text{o}}-{\:\text{T}}_{\text{a}\text{m}\text{b}}\right)}{{\text{A}}_{\text{S}\text{A}\text{H}\:}{\text{I}}_{\text{r}}}$$

*A*_*SAH*_ is the surface area of solar air heater needed.

The system efficiency of a solar dryer quantifies the effectiveness of utilizing input energy (solar radiation) for product drying. The equations for the system efficiency of a natural convection solar dryer and a forced convection system are as follows^8^:

For natural flow overall efficiency (η_on_):

11 $$\:{{\upeta\:}}_{\text{o}\text{n}}=\frac{{\text{M}}_{\text{r}\text{w}\:}{\text{h}}_{\text{f}\text{g}}}{{\text{I}}_{\text{r}}{\:\text{A}}_{\text{d}\:}+\:\text{Q}\text{u}}$$

where: m_rw_ is mass of water removed and it is given by the following Eq. ^25^:

12 $$\rm {M_{rw}}\;{M_i} - {M_f}$$

For forced flow, we are considering the power of the available auxiliary devices like a fan, pump, or any other external energy supplier.

The efficiency of the forced-type solar dryer:

13 $$\:{{\upeta\:}}_{\text{o}\text{f}}=\frac{{\text{M}}_{\text{r}\text{w}\:}{\text{h}}_{\text{f}\text{g}}}{{\text{I}}_{\text{r}}{\text{A}}_{\text{d}}+\:\text{F}\text{p}\:+\:\text{Q}\text{u}}$$

The value of latent heat of vaporization (h_fg_) of water, can be estimated using the following formula^26^:

14 $$\rm {h_{fg}} = ({\rm{ 2500.82}}-2.358\times{T_b}\, ln\:\frac{{\text{P}}_{\text{c}\text{r}}}{{10}^{5}}\:\frac{{(\text{T}}_{\text{c}}-{\text{T}}_{\text{p})}0.38}{{{(\text{T}}_{\text{c}\text{r}}-{\text{T}}_{\text{b}})}^{1.38}}$$

### Exergy analysis

The key parameters related to the exergy analysis of the solar air heater and drying chamber are discussed below.

### Exergy analysis of drying system

Exergy analysis of solar drying systems evaluates their efficiency and sustainability, offering insights into energy losses and opportunities for enhancement. The solar drying system comprises two main components: the solar air collector and the drying chamber. Analyses of both components are provided as follows.

### Exergy analysis of solar air heater

The exergy input (Ex_in, SAH_) and output (Ex_in, SAH_) associated with the solar air heater are defined in the following formulas^27^.

15 $$\rm{E{x_{in,{\rm{ }}SAH}}}\:=\left[1-\frac{{\text{T}}_{\text{S}\text{A}\text{H}\text{o}}}{{\text{T}}_{\text{s}}}\right]{\dot{\text{Q}}}_{\text{a},\text{S}\text{A}\text{H}}$$

where *T*_*s*_ represents the apparent temperature of the sun, which is approximately set at 6000 K, while $\:{\dot{Q}}_{a}$ denotes the solar energy absorbed by the absorber plate of the heater and is determined as follow^22^:

16 $$\:{\dot{Q}}_{a}={I}_{r}\left(\tau\:\alpha\:\right){A}_{{S}_{ah}}$$

Here, *α* represents the absorptivity of the copper plate, which is 0.95, and *τ* denotes the transmissivity of the window glass, which is 0.88. The exergy out flow of solar air heater is calculated using the following expression^24^:

17 $$\:\text{E}\text{x}_\text{o},\:\text{S}\text{A}\text{H}\:=\:{\dot{\text{m}}}_{\text{a}}\text{C}\text{p}\text{a}\left[\left({\text{T}}_{\text{S}\text{A}\text{H}\text{o}}-{\text{T}}_{{\text{S}}_{\text{a}\text{h}\text{i}}}\right)-{\text{T}}_{\text{S}\text{A}\text{H}\text{i}}\text{l}\text{n}\left(\frac{{\text{T}}_{\text{S}\text{A}\text{H}\text{o}}}{{\text{T}}_{\text{S}\text{A}\text{H}\text{i}}}\right)\right]$$

### Exergy efficiency

It serves as a gauge for the thermal system’s converted energy’s quality. It is the ratio of exergy outflow to exergy inflow as shown in following Eq. ^28^:

18 $${\rm {Exergy\, efficiency (}}{{\upeta\:}}_{Ex})=\frac{{\text{E}}_{\text{x}\text{o}}}{{\text{E}}_{\text{x}\text{i}}}=\frac{{\text{E}}_{\text{x}\text{i}}\:-\:{\text{E}}_{\text{x}\text{L}}}{{\text{E}}_{\text{x}\text{i}}}$$

### Exergy analysis of drying chamber

**Fig. 4:**
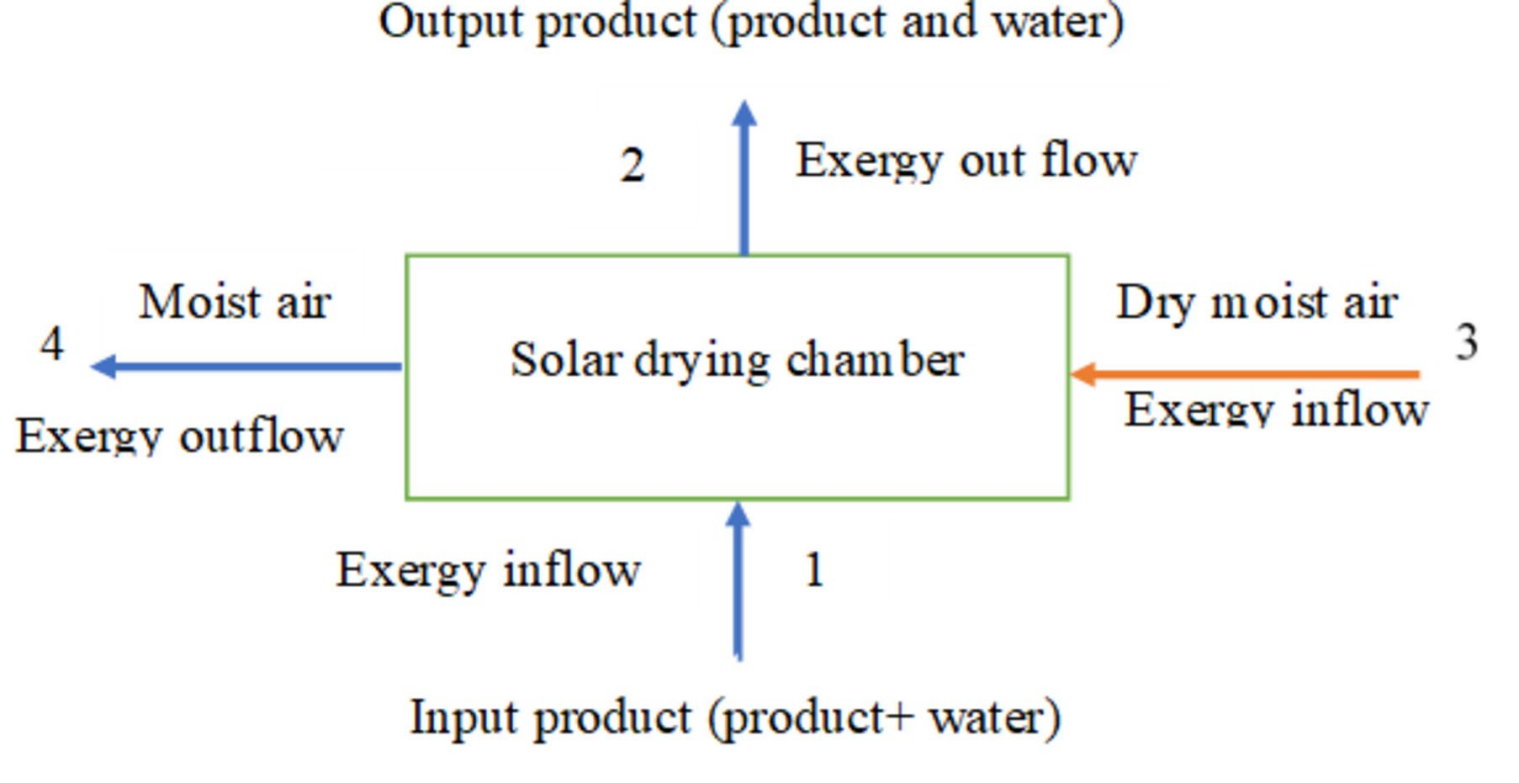
Energy and exergy inflow and outflow of drying system.

Exergy analysis of the solar drying system is conducted by assessing the exergy input, output, and losses of different components, including trays and the drying chamber^29^, as shown in Fig. 4.

The variance in flow exergy from the inlet to the outlet of the drying chamber equals to the combined total of thermal exergy loss and exergy destruction resulting from irreversibilities^7^. If the pressure between the inlet and out of the drying chamber is negligible the inflow exergy and outflow exergies at s steady-state by consecutive Eq^9^:

19 $$\:{\dot{\text{E}}}_{\text{x}\text{i}}\:$$=$$\:\:{\dot{\text{m}}}_{\text{a}}{\text{C}}_{\text{p}\text{a}}\left[({\text{T}}_{\text{c}\text{h}\text{i}}-{\text{T}}_{\text{a}\text{m}\text{b}})-{\text{T}}_{\text{a}\text{m}\text{b}}\text{l}\text{n}\left(\frac{{\text{T}}_{\text{c}\text{h}\text{i}}}{{\text{T}}_{\text{a}\text{m}\text{b}}}\right)\right]$$

20 $$\:{\dot{\text{E}}}_{\text{x}\text{o}}\:$$=$$\:\:{\dot{\text{m}}}_{\text{a}}{\text{C}}_{\text{p}\text{a}}\left[({\text{T}}_{\text{c}\text{h}\text{o}}-{\text{T}}_{\text{a}\text{m}\text{b}})-{\text{T}}_{\text{a}\text{m}\text{b}}\text{l}\text{n}\left(\frac{{\text{T}}_{\text{c}\text{h}\text{o}}}{{\text{T}}_{\text{a}\text{m}\text{b}}}\right)\right]$$

### Exergy analysis of the trays

If the pressure between the inlet and out, energies like kinetic, potential energies etc. are neglected inflow exergy and out flow exergies of the drying chamber trays can be calculated following expressions^30^:

### Exergy inflow of trays

21 $${\rm{Exergy\, inflow\, of\, bottom\, tray\,(E_{xT1in})=}}\:\:{\dot{\text{m}}}_{\text{a}}{\text{C}}_{\text{p}\text{a}}\left[\left({\text{T}}_{\text{t}\text{r}1}-{\text{T}}_{\text{a}\text{m}\text{b}}\right)-{\text{T}}_{\text{a}\text{m}\text{b}}\text{l}\text{n}\left(\frac{{\text{T}}_{\text{t}\text{r}1}}{{\text{T}}_{\text{a}\text{m}\text{b}}}\right)\right]$$

22 $${\rm{Exergy\, inflow\, of\, second\, tray\,(E_{xT2in})=}}\:{\dot{\:\text{m}}}_{\text{a}}{\text{C}}_{\text{p}\text{a}}\left[\left({\text{T}}_{\text{t}\text{r}2}-{\text{T}}_{\text{a}\text{m}\text{b}}\right)-{\text{T}}_{\text{a}\text{m}\text{b}}\text{l}\text{n}\left(\frac{{\text{T}}_{\text{t}\text{r}2}}{{\text{T}}_{\text{a}\text{m}\text{b}}}\right)\right]$$

23 $${\rm{Exergy\, inflow\, of\, third\, tray\,(E_{xT3in})=}}\:\:{\dot{\text{m}}}_{\text{a}}{\text{C}}_{\text{p}\text{a}}\left[\left({\text{T}}_{\text{t}\text{r}3}-{\text{T}}_{\text{a}\text{m}\text{b}}\right)-{\text{T}}_{\text{a}\text{m}\text{b}}\text{l}\text{n}\left(\frac{{\text{T}}_{\text{t}\text{r}3}}{{\text{T}}_{\text{a}\text{m}\text{b}}}\right)\right]$$

24 $${\rm{Exergy\, inflow\, of\, top\, tray\,(E_{xT4in})=}}\:{\dot{\text{m}}}_{\text{a}}{\text{C}}_{\text{p}\text{a}}\left[\left({\text{T}}_{\text{t}\text{r}4}-{\text{T}}_{\text{a}\text{m}\text{b}}\right)-{\text{T}}_{\text{a}\text{m}\text{b}}\text{l}\text{n}\left(\frac{{\text{T}}_{\text{t}\text{r}4}}{{\text{T}}_{\text{a}\text{m}\text{b}}}\right)\right]$$

### Exergy out flow of the trays

25 $${\rm{Exergy\, outflow\, of\, bottom\, tray\,(E_{xT1out})=}}\:{{\dot{\:\text{m}}}_{\text{a}}\text{C}}_{\text{p}\text{a}}\left[\left({\text{T}}_{\text{t}\text{r}2}-{\text{T}}_{\text{a}\text{m}\text{b}}\right)-{\text{T}}_{\text{a}\text{m}\text{b}}\text{l}\text{n}\left(\frac{{\text{T}}_{\text{t}\text{r}2}}{{\text{T}}_{\text{a}\text{m}\text{b}}}\right)\right]$$

26 $${\rm{Exergy\, outflow\, of\, second\, tray\,(E_{xT2out})=}}\:{{\dot{\:\text{m}}}_{\text{a}}\text{C}}_{\text{p}\text{a}}\left[\left({\text{T}}_{\text{t}\text{r}3}-{\text{T}}_{\text{a}\text{m}\text{b}}\right)-{\text{T}}_{\text{a}\text{m}\text{b}}\text{l}\text{n}\left(\frac{{\text{T}}_{\text{t}\text{r}3}}{{\text{T}}_{\text{a}\text{m}\text{b}}}\right)\right]$$

27 $${\rm{Exergy\, outflow\, of\, third\, tray\,(E_{xT3out})=}}\:{\dot{\:\text{m}}}_{\text{a}}{\text{C}}_{\text{p}\text{a}}\left[\left({\text{T}}_{\text{t}\text{r}4}-{\text{T}}_{\text{a}\text{m}\text{b}}\right)-{\text{T}}_{\text{a}\text{m}\text{b}}\text{l}\text{n}\left(\frac{{\text{T}}_{\text{t}\text{r}4}}{{\text{T}}_{\text{a}\text{m}\text{b}}}\right)\right]$$

28 $${\rm{Exergy\, outflow\, of\, top\, tray\,(E_{xT4out})=}}\:{\dot{\:\text{m}}}_{\text{a}}{\text{C}}_{\text{p}\text{a}}\left[\left({\text{T}}_{\text{c}\text{h}\text{o}}-{\text{T}}_{\text{a}\text{m}\text{b}}\right)-{\text{T}}_{\text{a}\text{m}\text{b}}\text{l}\text{n}\left(\frac{{\text{T}}_{\text{c}\text{h}\text{o}}}{{\text{T}}_{\text{a}\text{m}\text{b}}}\right)\right]$$

The specific heat of air (Cpa, in J/kg. K) is calculated using the following expression^31^:

29 $${C_{pa}}\;999.2{\rm{ }} + {\rm{ }}0.1434{T_{av}} + {\rm{ }}1.101\; \times {10^{ - 4}}{T_{av}}^2-6.758 \times {10^{ - 8}}{T_{av}}^3$$

where: T_av_ is the average of the ambient temperature and the boundary temperature of given system.

### Mathematical modeling and characterization of sample

As noted by Kaleta et al.^32^, a mathematical modelling is key aspect of drying technology which helps design engineers choose optimal operating conditions and properly size drying equipment to achieve desired performance. Several parameters are used to describe the behavior of a product during the drying process. Some of these are outlined in the sections below.

### Moisture content

To assess the moisture content (M_c_) of the samples on a wet basis throughout the day as they undergo the drying process^33^ and calculated using the following equation:

30 $${\rm{M_c}}=\frac{{M}_{w}-\:{M}_{d}}{{M}_{in}}$$

### Moisture ratio

The moisture ratio (MR) of the product is calculated as follow^21^:

31 $${\rm{MR}}\:\:=\frac{{M}_{t}-\:{M}_{e}}{{M}_{in}-{M}_{e}}$$

where: *M*_*t*_, *M*_*0*_, and *M*_*e*_ represent the moisture content at any given time during drying (kg of water per kg of dry matter), the initial moisture content (kg of water per kg of dry matter), and the equilibrium moisture content (kg of water per kg of dry matter), respectively.

Since the products were not continuously subjected to consistent relative humidity and temperature, and the values of *M*_*e*_ were significantly smaller than *M*_*t*_ or *M*_*0*_. As a result, the error caused by the simplification is insignificant^34^. Thus, Eq. (31) is simplified to Eq. (32).

32 $${\rm {MR}}\:\:=\frac{{M}_{t}}{{M}_{in}}$$

### Drying rate

The drying rate is a key parameter for assessing the efficiency of the drying process. It quantitatively indicates how swiftly the drying progresses and how effectively it reaches the target moisture level for preservation^33^ and calculated as follow:

33 $${\rm{DR}}=\:\:\:\frac{{\text{M}}_{\text{w}}-{\:\text{M}}_{\text{d}}}{\text{T}\text{o}\text{t}\text{a}\text{l}\:\text{d}\text{r}\text{y}\text{i}\text{n}\text{g}\:\text{t}\text{i}\text{m}\text{e}\:}$$

### Thin layer drying models

Food materials are inherently heterogeneous in their structure and chemical composition. Thus, the drying kinetics models can characterize the average moisture content of the food product, without accounting for the heterogeneity within the material^35^. Some of the thin layer models developed by various scholars to describe the behavior of agricultural products are listed in Table 2, where: *MR* = Moisture ratio (-),t = time (hours), *k*_*1*_, *k*_*2*_, *k*_*3*_ = drying constants (1/hour) and *a*,* b*,* c*,* d*,* e*,* f*,* g* and *n* = empirical parameters of constants (-).

**Table 2:** Thin layer drying models^36^.

S/*N*	Model name	Formula
1	Newton	MR$\:\:=$ exp (− *k t*)
2	Wang and Singh models	MR = 1 + *a t* + *b t*^2^
3	Page model	MR = exp$\:\left(-{t}^{n}\right)$
4	Modified page model	MR = exp$\:\left(-{\left(k\:t\right)}^{n}\right)$
5	Logarithmic	MR = *a* exp (− *k t*) + c
6	Two-term	MR = $\:a\:exp\left(-kt\right)+b\:exp\left({-k}_{1}t\right)$
7	Two-term exponential model	MR$\:=a\:exp\left(-kt\right)+\left(1-a\right)exp\left(kat\right)$
8	Weibull distribution	MR $\:=a-b\:exp\left(-\left(k\:{t}^{n}\right)\right)$
9	Handerson and Pabis	MR$\:\:=a\:exp\left(k\:t\right)$
10	Midilli et al.	MR$\:=a\:exp\left(-k{\:t}^{n}\right)+b\:t$

The following non-linear regression analysis was performed to assess the goodness of fit of the tested mathematical models. Key metrics such as the coefficient of determination (R²) and the reduced chi-square (χ²), root mean square error (RMSE) and Um squared error (SSE) were used to compare the predicted and experimental values for each model. the statistical metrics used to evaluate the thin layer models are listed in table 3.

**Table 3:** Statical metrices^37^.

Name of statistical tool	Formula
Coefficient of determination (R^2^)	R^2^$\:\:=\frac{n\sum\:_{i=1}^{n}{x}_{i}{y}_{i}-\left(\sum\:_{i=1}^{n}{x}_{i}\right)\left(\sum\:_{i=1}^{n}{y}_{i}\right)}{\sqrt{\left[n\sum\:_{i=1}^{n}{x}_{i}^{2}-{\left(\sum\:_{i=1}^{n}{x}_{i}\right)}^{2}\right]}\sqrt{\left[n\sum\:_{i=1}^{n}{y}_{i}^{2}-{\left(\sum\:_{i=1}^{n}{y}_{i}\right)}^{2}\right]}}$
chi-square (X^2^)	X^2^$\:\:=\frac{\sum\:_{i=1}^{n}{\left({x}_{i}-{y}_{i}\right)}^{2}}{n-C}$
Root Mean Square Error (RMSE)	RMSE$\:\:=\sqrt{\sum\:_{i=0}^{n}\frac{{\left({x}_{i}-{y}_{i}\right)}^{2}}{n}}$
Sum squared error (SSE)	SSE$\:\:=\sum\:_{i=1}^{n}{\left({x}_{i}-{y}_{i}\right)}^{2}$

## Result and discussion

The data were measured every minute, except for the mass of the samples, which was measured every hour. However, for better clarity and visualization, the data were averaged into 30-minute intervals.

### Calculated values

The calculated properties of air like specific heat capacity of (C_p.a._) of 1.0084 KJ/kg.K and air density (ρa) of 1.159 kg/m³. The latent heat of vaporization (h_fg_) was estimated 229.855 KJ/kg.

### Variation of solar radiation

**Fig. 5:**
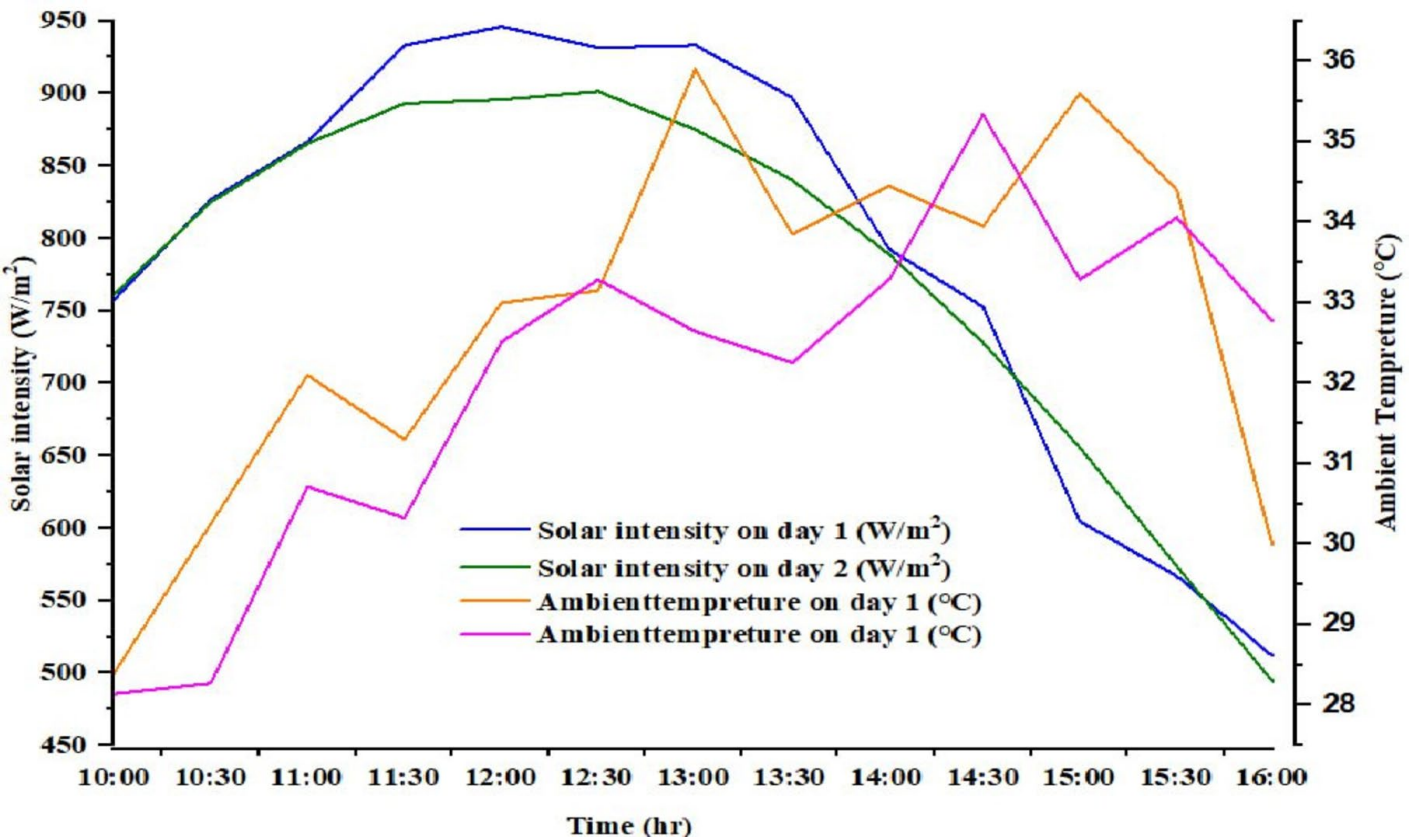
Ambient and solar radiation of the site under study during the experiment.

The experiment was conducted between 10:00 AM and 4:00 PM. The experiments were conducted during clear sky conditions. The daily variations in radiation intensity and ambient temperature are illustrated in Fig. 5. Thus, Solar radiation was characterized by a rise in the morning, a peak around midday, and a gradual decline in the afternoon. On day 1, radiation levels were consistently higher than on day 2, with a particularly noticeable difference in the rate of decline after 12:30 PM, culminating in a significant drop by 4:00 PM. Additionally, day 1 recorded higher ambient temperatures and radiation levels compared to day 2. The average ambient temperatures for day 1 and day 2 were 32.79 °C and 29.55 °C, respectively, while the average solar radiation intensities were 793.84 W/m² and 774.76 W/m².

### Humidity analysis

**Fig. 6:**
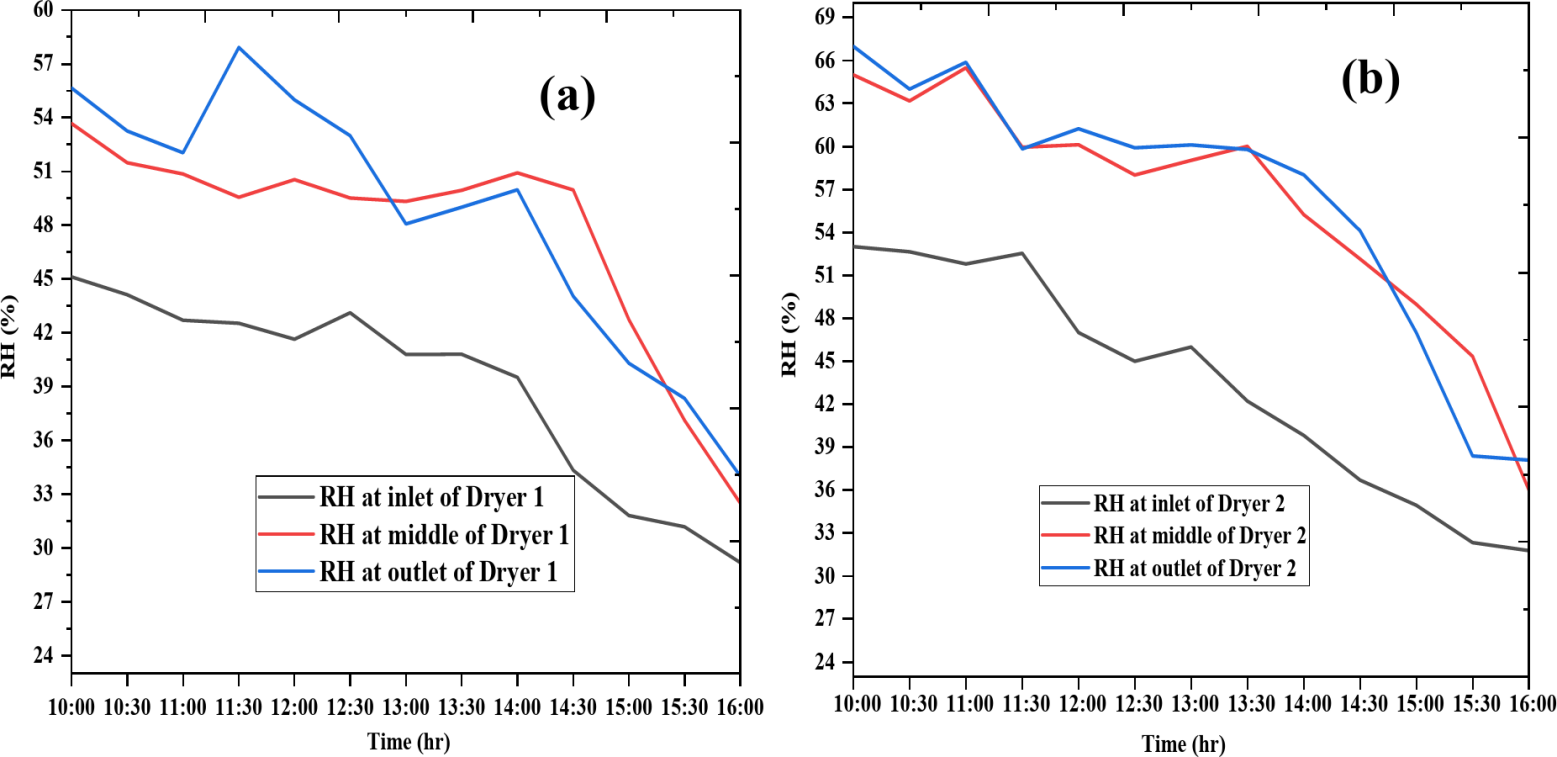
Humidity variation of Dryer 1 (**a**) and Dryer 2(**b**) on the first day.

**Fig. 7:**
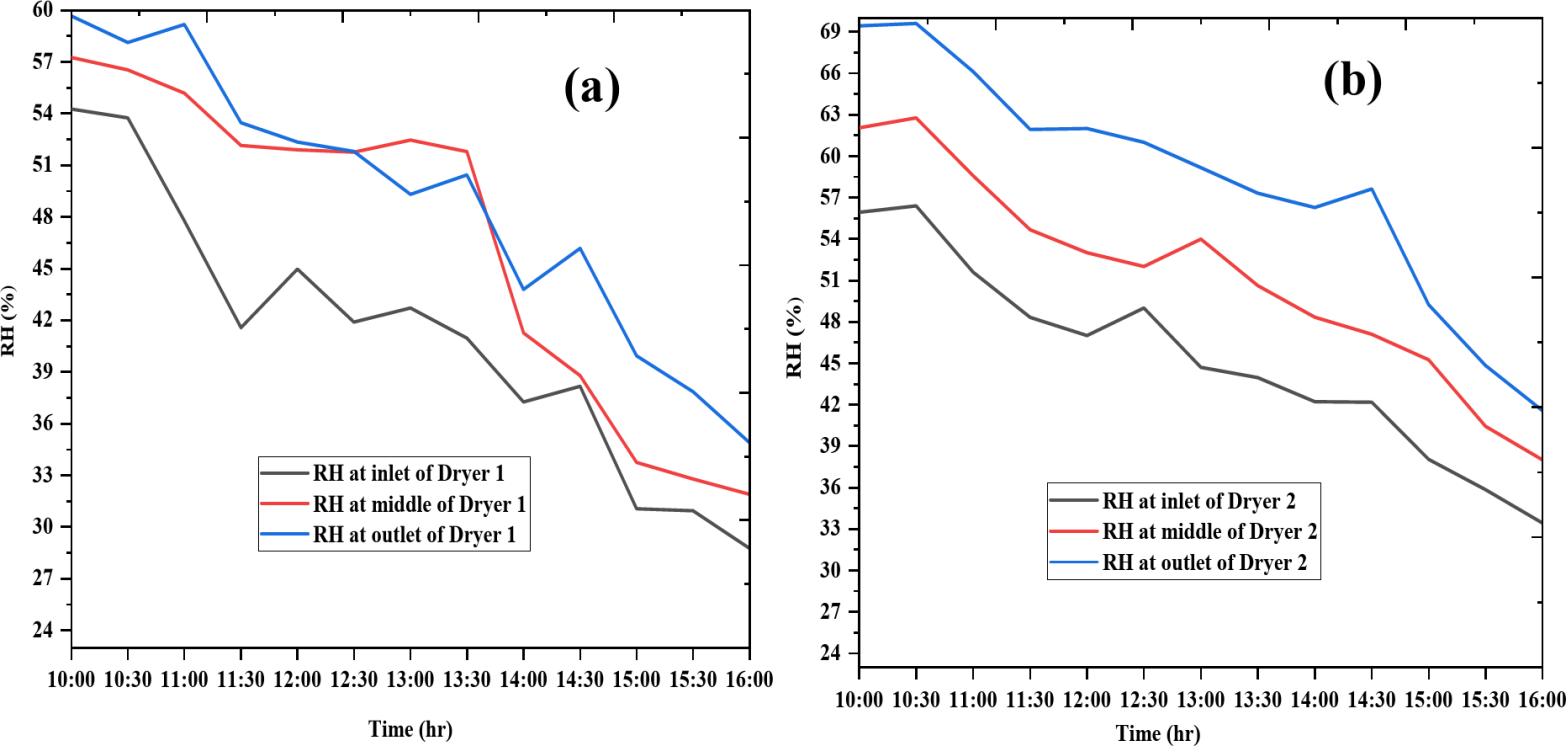
Humidity variation of Dryer 1(**a**) and Dryer 2(**b**) on the second day.

Humidity analysis of the drying chamber is presented in Fig. 6 for day 1 and Fig. 7 for day 2. Humidity levels were highest at the outlet, followed by the middle, and lowest at the inlet of the drying chamber. On day 1, the average humidity values for Dryer 1 at the inlet, middle, and outlet were 39.0%, 47.6%, and 48.5%, respectively, while on day 2, they were 40.8%, 46.7%, and 49.2%. For Dryer 2, the average humidity values on day 1 were 43.1%, 56.9%, and 56.6% at the inlet, middle, and outlet, respectively, and on day 2, they were 45.2%, 51.3%, and 58.3%. Dryer 1 demonstrated superior performance due to its lower outlet humidity, indicating more effective drying. Overall, both dryers showed a consistent decrease in humidity across all sections of the chamber from morning to afternoon, confirming efficient drying performance.

## Energy and exergy analysis

### Energy efficiency evaluation of the drying chambers and solar air heaters

**Fig. 8:**
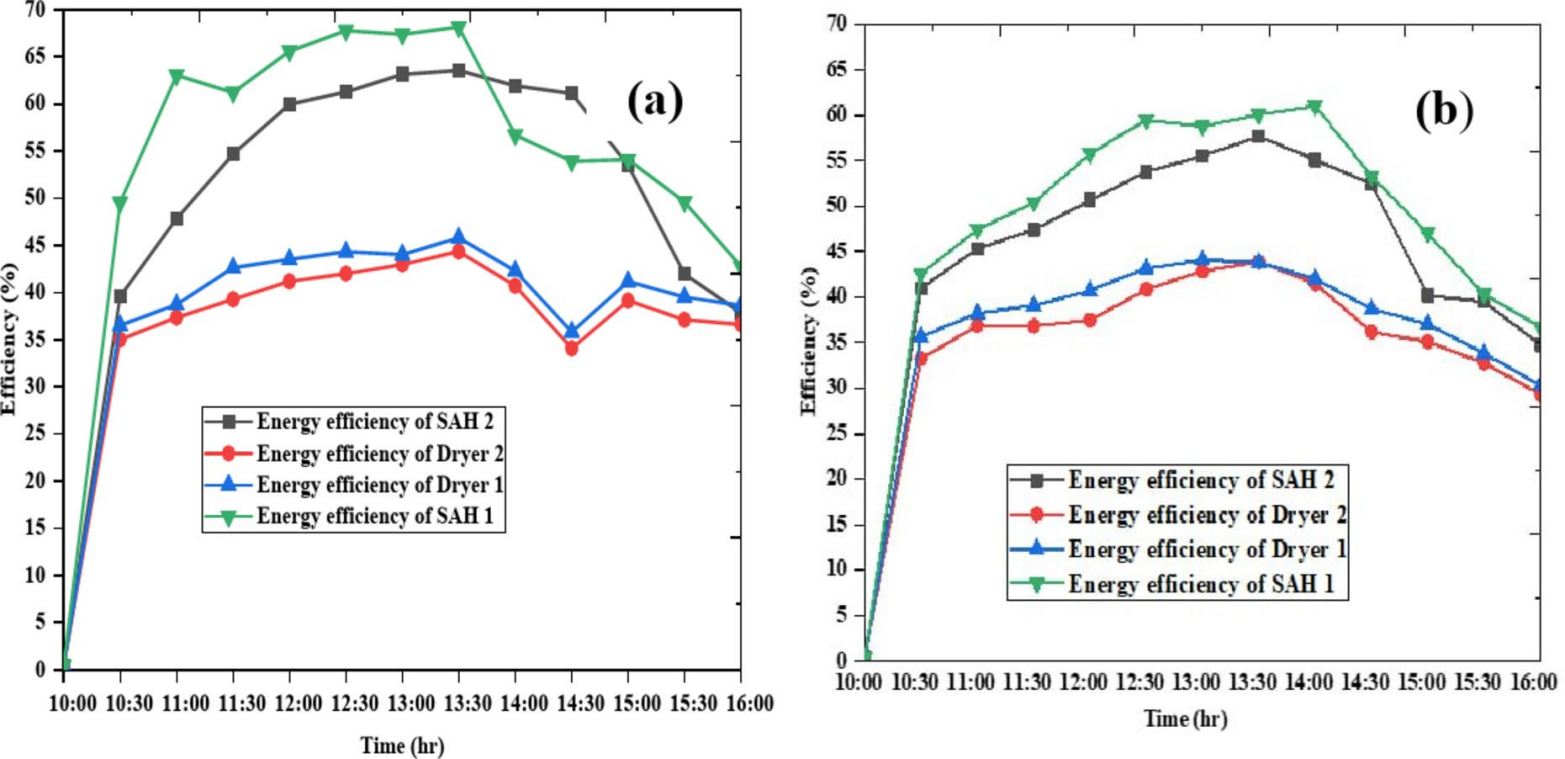
Efficiency of the drying chambers and solar air heaters on day 1(**a**) and on day (**b**)

The efficiency of a solar air heater and drying chamber is a critical factor in evaluating the performance of solar drying systems. Figure 8 provided shows the efficiency of two dryers and two solar air heaters over time during the day 1 and day 2. So, as shown in Fig. 8 the efficiency of both dryers and days solar air heaters experiences a significant increase from 10:00 AM to approximately 1:30 PM, reaching its peak during midday when solar radiation is at its highest. Following 1:30 PM, efficiency gradually declines as solar radiation diminishes in the afternoon and evening. This version emphasizes the key points while ensuring a smooth flow of information.

### Exergy analysis of solar air heaters

The exergy inflow and outflow for both solar air heaters over the two days are depicted in Fig. S1 (a) and (b), respectively. Both systems exhibit similar trends, SAH 1 consistently outperforms SAH 2 by a small margin. The data underscores the importance of optimizing system design and positioning to maximize exergy availability, particularly during peak solar radiation hours. The first solar air heater (SAH 1) was positioned slightly higher than the second solar air heater (SAH 2) as shown in Fig. 1. Thus, the findings emphasize the importance of strategic placement and design in optimizing the efficiency of solar air heating systems. Further studies could explore additional factors, such as orientation and tilt angle, to identify the most effective configuration for maximizing energy and exergy efficiency.

### Exergy inflow and out flow of the drying chambers

The variation of the exergy inflows and out flow of both dryers over time is shown in Fig.S2. These results indicate that while both dryers exhibit a decrease in efficiency over time, dryer 1 demonstrates a higher efficiency compared to Dryer 2, suggesting that it is more effective in converting inflow exergy into useful output during the observed period.

### Exergy inflow of the trays of both dryers on day 1

**Fig. 9:**
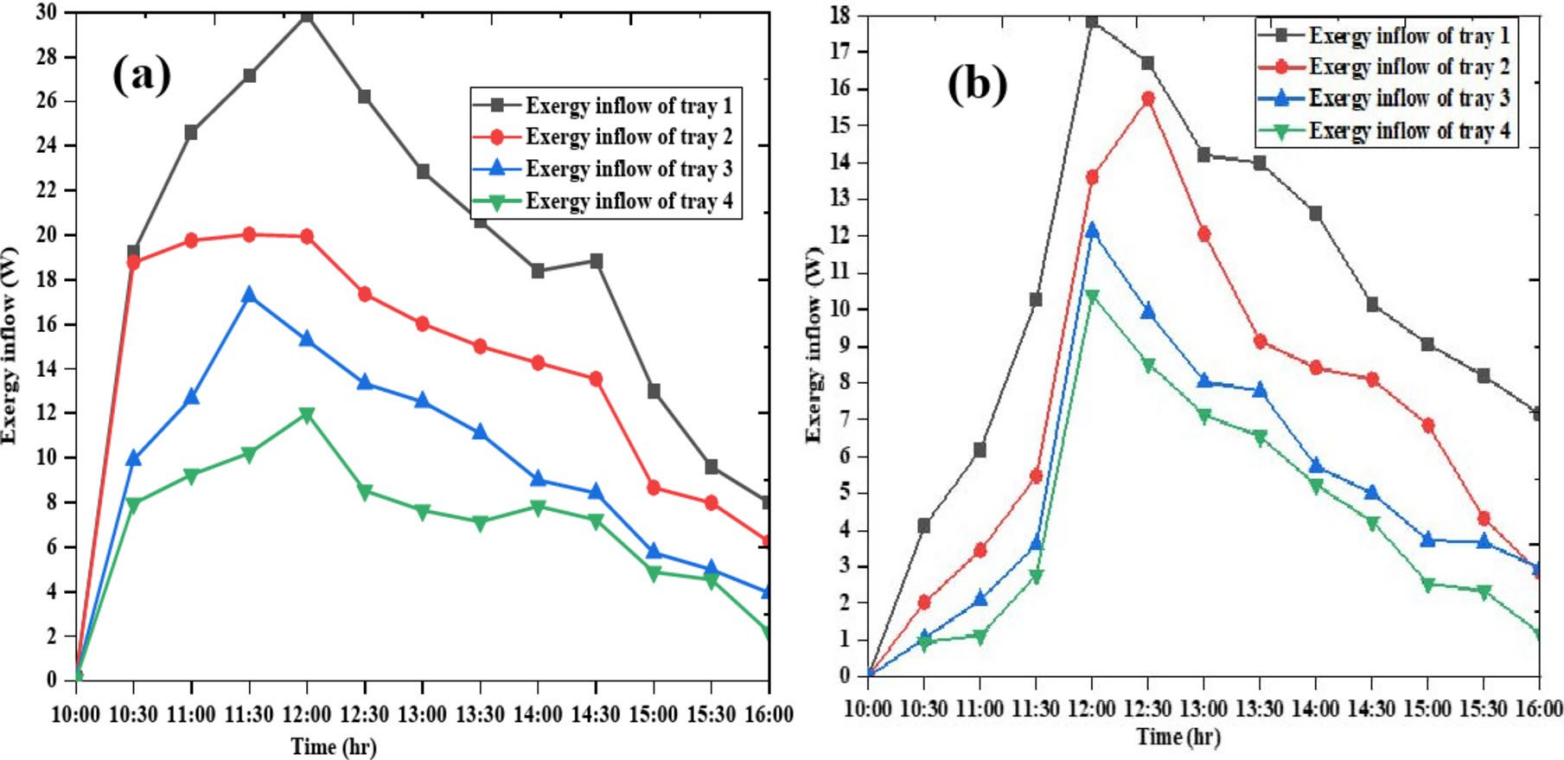
Exergy inflow of the trays of Dryer 1(**a**) and Dryer 2 (**b**) on day 1.

**Fig. 10:**
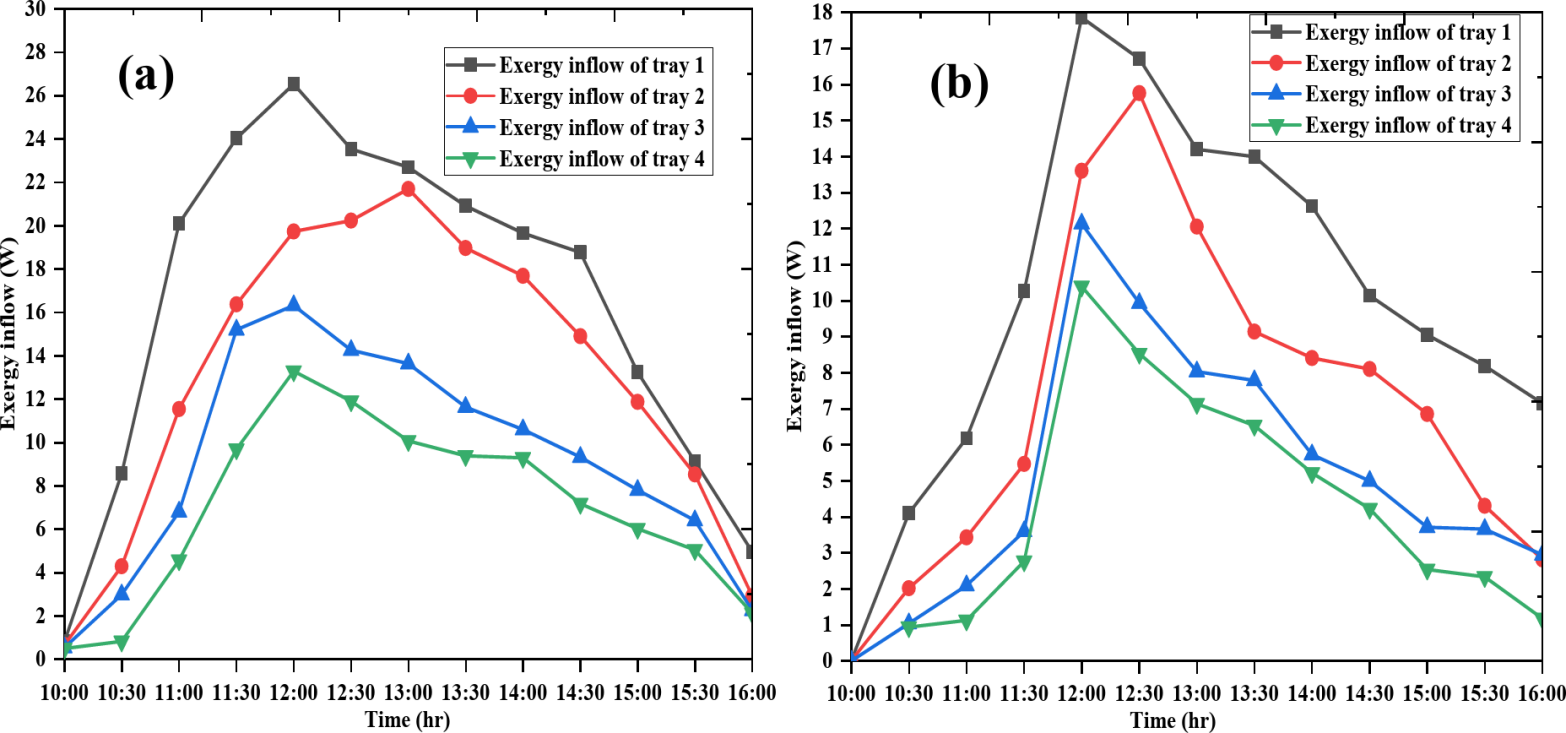
Exergy inflow of the trays of Dryer 1(**a**) and Dryer 2(**b**) on day 2.

The exergy inflow of the trays for both days is shown in Figs. 9 and 10, respectively. As shown in the indicated Figures the exergy in flow in both days and both dryers a demonstrate significant variations over time. The exergy flow of tray 1consistently exhibits the highest exergy values, indicating it is the most efficient or possesses the highest useful work potential among the four systems analyzed. In contrast, tray 4 shows the lowest exergy inflow values, suggesting it is the least efficient in terms of useful work output. The peak exergy values for most systems occur around 12:00 PM, which aligns with typical solar thermal systems’ performance due to maximum solar radiation and energy input at this time.

### Exergy out flow analysis of trays of both dryers on day 1 and day 2

Figures S3 (a and b) and S4 (a and b) illustrate the exergy outflow of trays for both dryers over two days. Tray 1 exhibits the highest exergy outflow, while tray 4 shows the lowest. This trend corresponds with the exergy inflow patterns, where tray 1 receives the most energy and tray 4 the least. Tray 1 demonstrates the highest exergy outflow throughout the day, indicating that tray 1 is the most efficient in utilizing input energy for drying. Tray 2 follows a similar pattern but with reduced values, suggesting lower efficiency compared to tray 1. The trays 3 and 4 display significantly lower exergy outflow, with tray 4 being the least efficient, consistent with its minimal exergy inflow.

### Exergy efficiency of the solar air heaters and drying chamber

**Fig. 11:**
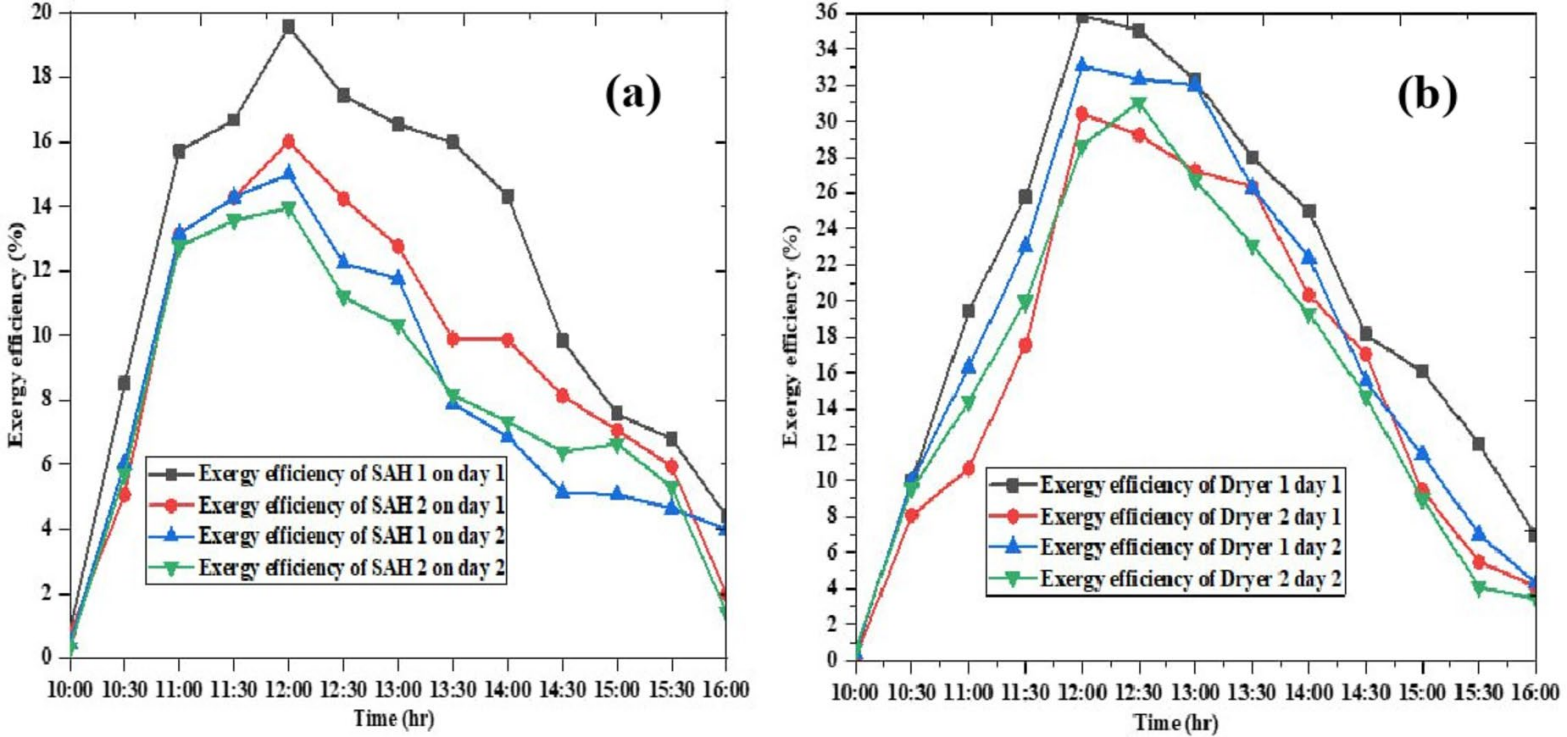
Exergy efficiency of the solar air heaters (**a**) and drying chambers (**b**).

As illustrated in Fig. 11(a) both solar air heaters (SAH 1 and SAH 2) achieved their highest exergy efficiencies around 12:00 on both days. Similarly, on Fig. 11(b) both dryers achieved their highest exergy efficiency around 12:00. The average efficiency of SAH 1 and SAH 2 were 47.18% and 44.19%, respectively, with the dryers’ average efficiencies at 35.9% and 34.4%. The average exergy efficiency of SAH 1 and SAH 2 on day 1 were 11.85% and 9.15%, respectively, while the corresponding dryers had average exergy efficiencies of 20.37% and 16.00%. On day 2, the average exergy efficiency of dryer 1 was 17.93%, and dryer 2’s was 15.73%. In general, the performance of the drying components dryers was heavily influenced by solar radiation, with efficiencies peaking around midday and declining in the morning and evening.

### Moisture content evaluation

**Fig. 12:**
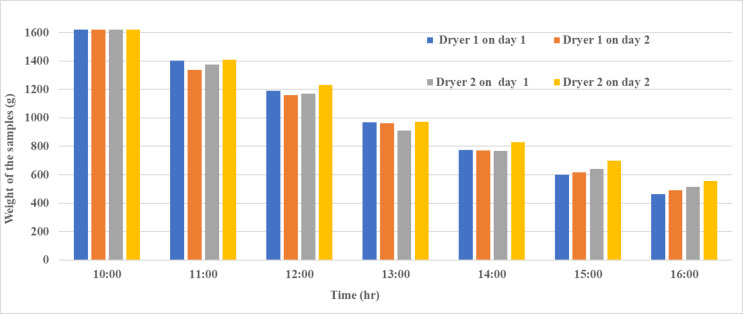
Moisture analysis of the given samples.

During the drying analysis, each tray was loaded with 405 g, totaling 1620 g across the four trays. The moisture loss of the samples is illustrated in Fig. 12. The most notable mass reduction was observed between 10:00 and 13:00 across all dryers and days. Overall, Dryer 1 demonstrated consistently higher mass reduction rate compared to Dryer 2 on both days.

### Moisture ratio analysis and drying curve

**Fig. 13:**
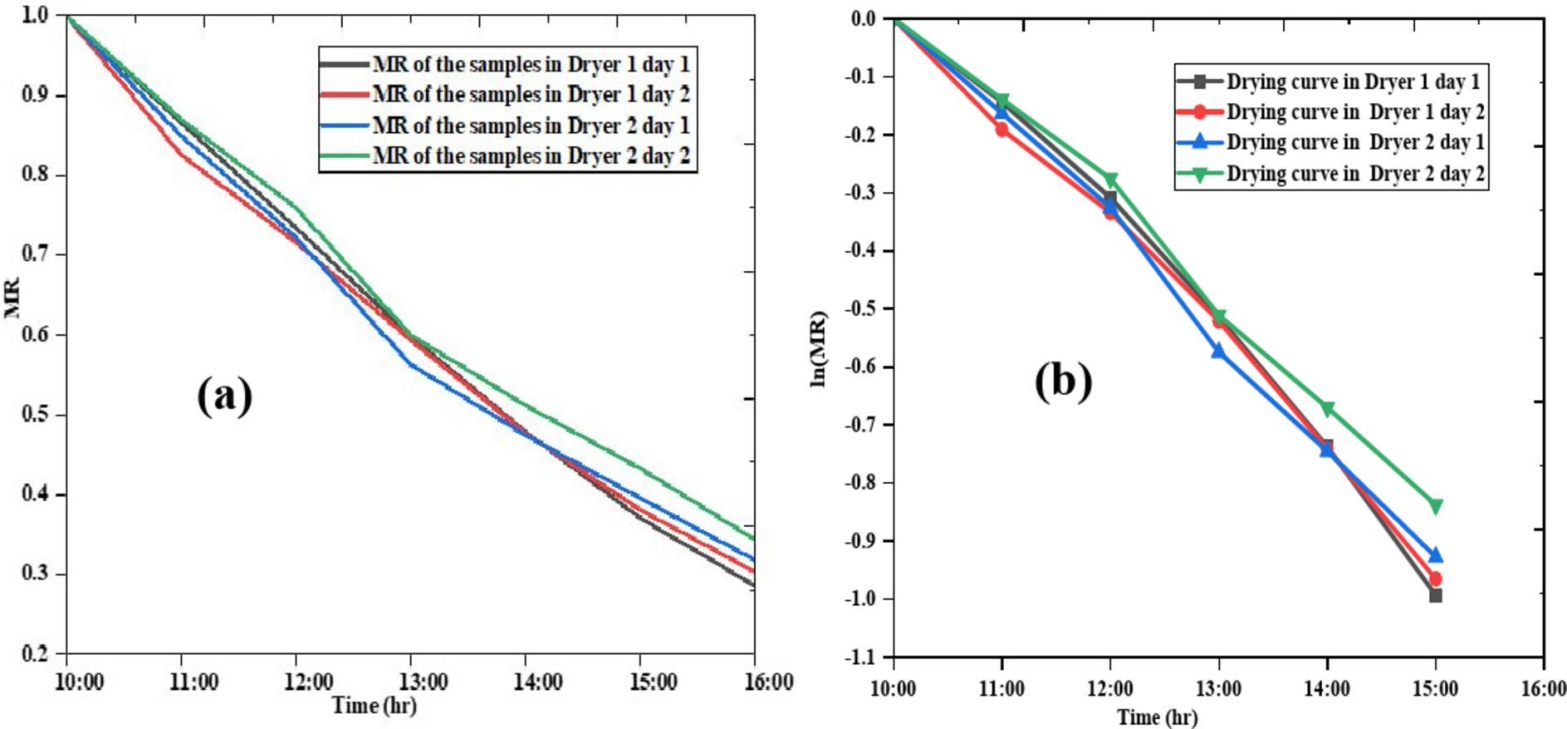
Moisture ratio of the sample for both dryer and drying curve of given apple slices.

The moisture ratio (MR) versus time is illustrated in Fig. 13(a) and the natural logarithm of MR versus time, which is the drying curve is shown in Fig. 13(b). The moisture ratio decreases continuously with drying time. Overall, both dryers effectively reduce moisture content across both days, with MR values progressively decreasing from morning to afternoon. These observations dryer1 have a slight efficiency advantage, particularly on the first day. This analysis provides insights that could be used to optimize drying parameters, especially for dryer 2, to ensure uniform drying across multiple days. In the case of drying curve in initial stages and gradual tapering. The drying of apple slices occurs during a falling rate period, with no occurrence of a constant rate period.

### Selecting the best fitting model

To select the most suitable thin-layer model for drying apple slices in both dryers, ten mathematical models were evaluated. Statical metrices namely coefficient of determination (R²), root mean square error (RMSE), chi-square (x²), and sum of squared errors (SSE) were employed to identify the best-fitting mode. Based on the metrices Midilli et al. model, along with the logarithmic and two-term models, provided the best fit across both drying days. The detailed numerical analysis is provided in Tables S1 to S3.

Based the Table S1 results the thin layer drying model for golden in dryer 1 in day 1 is formulated as follow:

Midilli et al.:

34 $${\rm{MR}}=2.82\:\text{e}\text{x}\text{p}\left(-5.02{\:\text{t}}^{1.80}\right)+0.0029\text{t}$$

Logarithmic:

35 $$\rm MR= 9.2\,exp\,( - 5.29\,t)$$

Two terms:

36 $${\rm{MR=}}\:4.7\:\text{e}\text{x}\text{p}\left(-3.97\:\text{t}\right)+0.0010\:\text{e}\text{x}\text{p}\left(-1.0970\:\text{t}\right)$$

## Conclusion and recommendations

The study presents an evaluation of the energy and exergy assessments of two drying chambers with different shapes over a two-day period. Apple was used as the sample for drying, and the drying behavior of this sample was also investigated. The research reveals key insights into solar drying system performance. Energy and exergy efficiencies are influenced by solar radiation and ambient temperatures. Humidity levels in the drying chamber were highest in the bottom tray and decreased upwards. The solar air collector’s positioning and drying chamber design are crucial for system performance. Increased efficiency of solar heaters boosts dryer performance. The chamber shape has a minor but notable impact on overall efficiency. The Midilli and Kucuk model, logarithmic model, and two-term model best fit the data.

For future research, it is advisable to employ simulations such as computational fluid dynamics (CFD) and artificial neural networks (ANN) to optimize the performance and improve the efficiency of dryers. Furthermore, investigating the economic feasibility of drying chambers in relation to their visibility in the global market presents a promising opportunity.

## Electronic supplementary material

Below is the link to the electronic supplementary material.

Supplementary Material 1

Supplementary Material 2

## Data Availability

The data sets used and analyzed during the current study are available from the corresponding author upon reasonable request.

## Abbreviations

A_OUT_: Radiometric analog voltage output
A_SAH_: Area of the solar dryer (m^2^)
Ad: Area of the dryer (m^2^)
C_d_: Discharge coefficient ranges 0.6 to 0.9 for most orifices
cDAQ: Compact data acquisition
C_p.a._: Specific heat of air (kJ/kg.K)
Dch: Drying chamber
DR: Drying rate (kg/hr)
Ex: Exergy (kJ/kg)
E_xi_: Exergy inflow (kJ/kg)
Exo: Exergy outflow(kJ/kg)
F_p_: Fan power (Watt)
g: Gravitational acceleration, (m/s^2^); gram
gc: Dimensional conversion constant
hr: Hour of drying (hr)
I_r_: The amount of radiation striking the collector’s plane (W/m^2^)
ṁ: Mass flow rate, (kg/s)
M_f_: Final mass of the sample at end of the time (wb)
M_in_: Initial mass of the sample (wb)
M_d_: Mass of the dried sample at time t (wb)
M_t_: Instantaneous moisture content (wb)
M_w_: Mass of the wet sample at time t (kg)
Om: Orifice meter
P: Power (W)
RH: Relative humidity (%)
RTD: Resistance temperature detector
SAH: Solar air heater
T: Temperature (°C)
T_amb_: Ambient temperature (°C)
T_b_: Temperature at which water boils under standard atmospheric pressure (°C)
Tcr: Critical temperature of water (377 °C)
Tchi: Temperature entering to the drying chamber (°C)
Tcho: Temperature leaving the drying chamber (°C)
T_saho_: Temperature leaving the solar air heater (°C)
T_r_: Tray
V_DD_: Power supply voltage
W: Watt
x: Experimental (observed) data
y: Predicted (calculated) data

## Subscripts

a: Air
amb: Ambient
ch: Drying chamber
in: Inlet
L: Loss
o: Outlet
t: Total
wb: Wet basis
