# Supplementary material for: Mathematical modelling of golden apple drying and performance evaluation of solar drying systems using energy and exergy approach
Source: Sci Rep. 2025 Mar 6;15:7805. doi: 10.1038/s41598-025-92133-2 (PMC11885661; doi:10.1038/s41598-025-92133-2)
Supplement: Supplementary file 1 — Supplementary Material 1 [file 41598_2025_92133_MOESM1_ESM.docx]

**Supplementary tables**

Table S1. Statistical results of drying curve models for apple slice moisture data dryer 1in day1

| Model Name | Constants | R^2^ | RMSE | X^2^ | SSE |
| --- | --- | --- | --- | --- | --- |
| Newton | K=1.1 | 0.999249 | 0.07682 | 0.5306 | 0.28919 |
| Wang and Singh | a=0, b=0 | - | 0.5975 | 0.7142 | 1.4284 |
| Page | n=0 | - | 1.3977 | 7.8150 | 31.2600 |
| Modified page | k=1, n=1 | 0.9991 | 0.2611 | 0.13637 | 0.2727 |
| **Logarithmic** | **a=9.2, k=5.29, c=0** | **0.9920** | **0.0453** | **0.0061** | **0.0184** |
| **Two-term** | **a=4.7, k_1_=3.97, b=0.0010, k_2_=1.0970** | **0.9966** | **0.0465** | **0.0086** | **0.0347** |
| Two-term  exponential | a=2.7, k=0.11 | 0.9969 | 0.2444 | 0.1195 | 0.2390 |
| Weibull distribution | a=0.6, b=0,  k=0.41, n=0.23 | 0 | 0.3258 | 0.0096 | 0.4246 |
| Handerson and Pabis | a=0.6,  k=0 | - | 0.0954 | 0.0892 | 0.4463 |
| **Midilli and Kucuk** | **a=2.82, b=0.0029,**  **k=5.02, n=1.80** | **0.9969** | **0.03105** | **0.00385** | **0.0154** |

Table S2. Statistical results of drying curve models for apple slice moisture data dryer 1day 2

| Model Name | Constants | R^2^ | RMSE | X^2^ | SSE |
| --- | --- | --- | --- | --- | --- |
| Newton | K=1.1 | 0.9974 | 0.4980 | 0.2480 | 0.2480 |
| Wang and Singh | a=0, b=0 | - | 0.5955 | 0.7094 | 1.4189 |
| Page | n=0 | - | 5.6015 | 31.3778 | 31.3778 |
| Modified page | k=1, n=1 | 0.9972 | 0.2451 | 0.05842 | 0.24045 |
| **Logarithmic** | **a=9.2, k=5.29, c=0** | **0.9957** | **0.0399** | **0.0047** | **0.0143** |
| **Two-term** | **a=4.7, K_1_=3.97, b=0.0010, k_2_=1.0970** | **0.9985** | **0.03771** | **0.0056** | **0.0293** |
| Two-term  exponential | a=2.7, k=0.11 | 0.9901 | 0.0693 | 0.0408 | 0.2353 |
| Weibull distribution | a=0.6, b=0, k=0.41, n=0.23 | 0 | 0.0954 | 0.1487 | 0.4463 |
| Handerson and Pabis | a=0.6,  k=0 | - | 0.0954 | 0.0892 | 0.4463 |
| **Midilli and Kucuk** | **a=2.82, b=0.002,**  **k=5.02, n=1.80** | **0.9985** | **0.0289** | **0.0033** | **0.0134** |

Table S3. Statistical results of drying curve models for apple slice moisture data dryer 2 day 1

| Model Name | Constants | R^2^ | RMSE | X^2^ | SSE |
| --- | --- | --- | --- | --- | --- |
| Newton | K=1.1 | 0.9944 | 0.5002 | 0.2502 | 0.2502 |
| Wang and Singh | a=0, b=0 | - | 0.5910 | 0.6986 | 1.3972 |
| Page | n=0 | - | 5.5915 | 31.2649 | 31.2649 |
| Modified page | k=1, n=1 | 0.994 | 0.2457 | 0.1208 | 0.2416 |
| **Logarithmic** | **a=9.2, k=5.29, c=0** | **0.9971** | **0.0412** | **0.0050** | **0.0050** |
| **Two-term** | **a=4.7, k_1_=3.97, b=0.0010, k_2_=1.0970** | **0.9987** | **0.0390** | **0.0061** | **0.0337** |
| Two-term  exponential | a=2.7, k=0.11 | 0.9936 | 0.2282 | 0.1041 | 0.2083 |
| Weibull distribution | a=0.6, b=0, k=0.41, n=0.23 | 0 | 0.1548 | 0.0959 | 0.3838 |
| Handerson and Pabis | a=0.6, k=0 | - | 0.3097 | 0.079402 | 0.1919 |
| **Midilli and Kucuk** | **a=2.82, b=0.0029,**  **k=5.02, n=1.80** | **0.9989** | **0.0308** | **0.0038** | **0.0152** |

**Supplementary Figures**


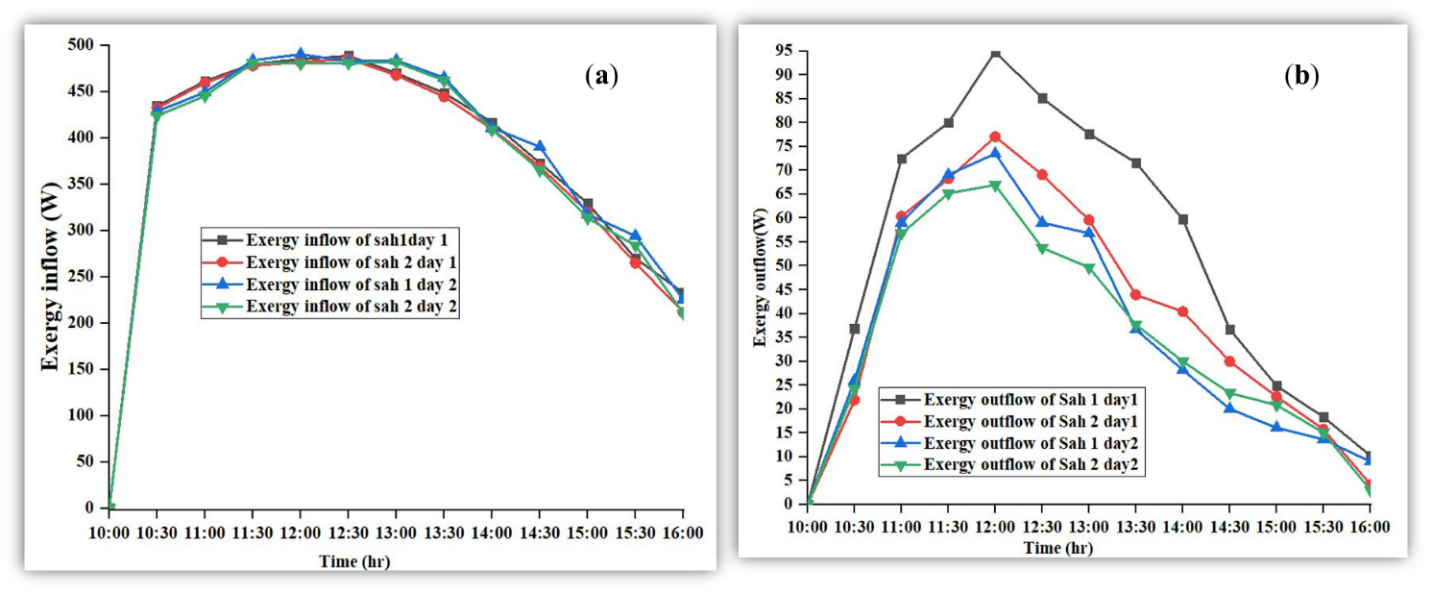


Fig.S1. Exergy inflow of (a) and exergy out flow (b) of the solar air


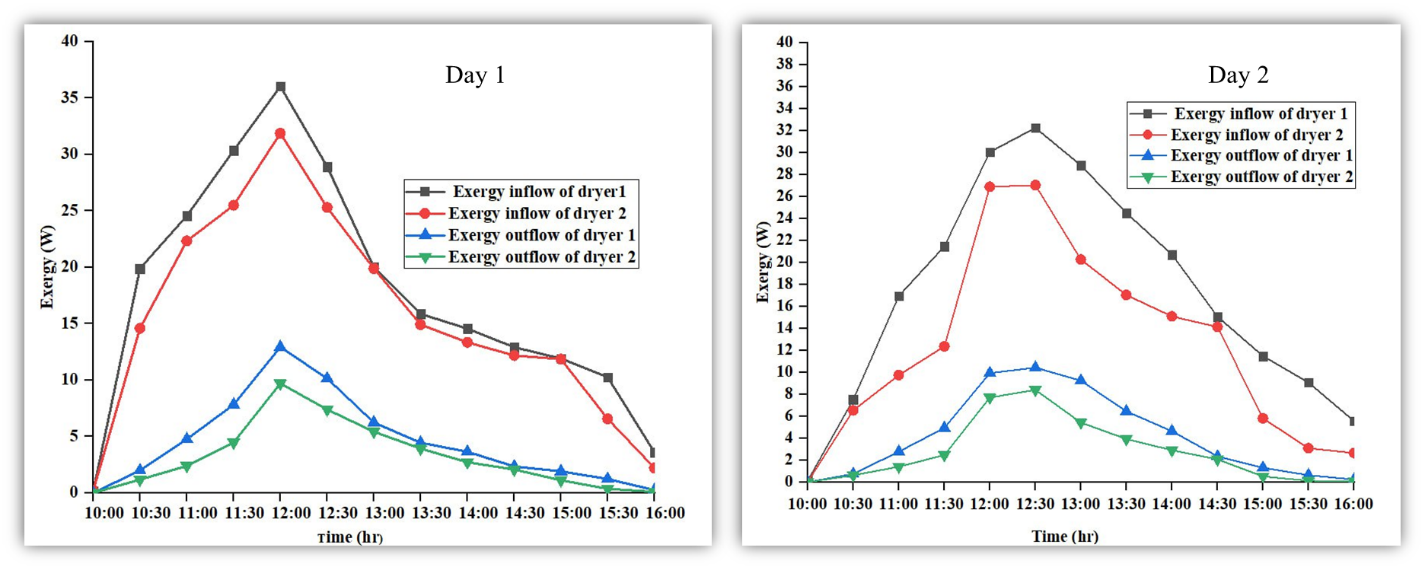


Fig.S2. Exergy analysis of the drying chamber in both days


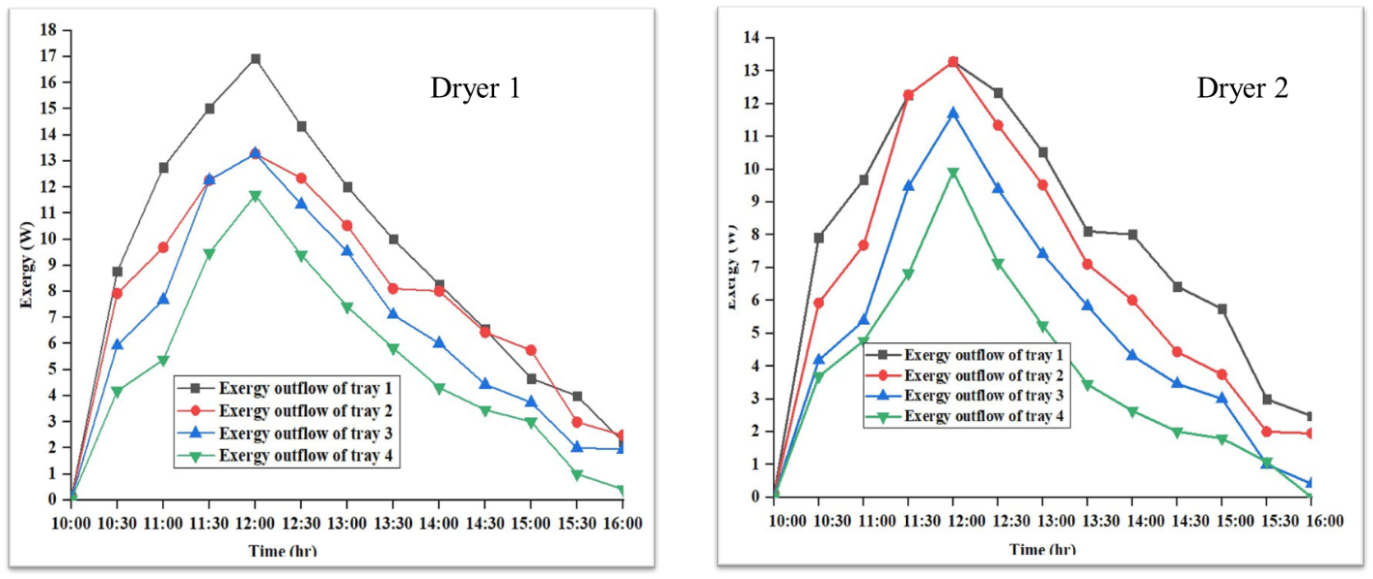


Fig.S3 Exergy out flow of trays in the dryer in day 1


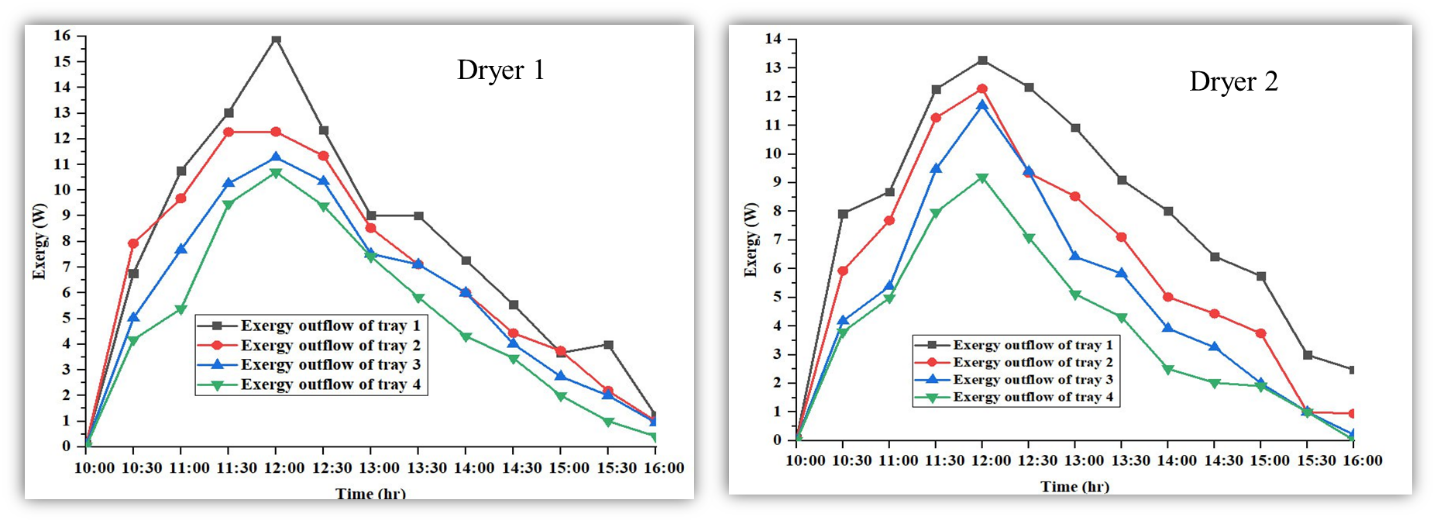


Fig.S4. Exergy out flow of trays in the dryer in day 2
